# Ectopic maxillary third molar associated with an odontogenic keratocyst in the maxillary Sinus presenting as refractory unilateral sinonasal symptoms: a case report with implications for early dental diagnosis

**DOI:** 10.3389/froh.2026.1866386

**Published:** 2026-07-24

**Authors:** Tao Weng, Mengjie Dong, Xiaoyin Gu, Yanyan Sun

**Affiliations:** 1 Department of Oral and Maxillofacial Surgery, Hangzhou Stomatology Hospital, Hangzhou, Zhejiang, China; 2 School of Medicine, Hangzhou Normal University, Hangzhou, Zhejiang, China; 3 Department of Stomatology, Zhejiang Hospital of Integrated Traditional Chinese and Western Medicine, Hangzhou, Zhejiang, China

**Keywords:** cone-beam computed tomography, ectopic tooth, maxillary sinus, odontogenic keratocyst, third molar

## Abstract

**Background:**

Ectopic teeth in the maxillary sinus are relatively rare, most commonly seen in the third molars, often accompanied by odontogenic cysts and presenting with symptoms similar to chronic sinusitis, which are easily misdiagnosed or overlooked.

**Case(s):**

A 24-year-old female patient presented with purulent nasal discharge and recurrent swelling of the right side of the face for 2 years. Despite multiple courses of antibiotic treatment, her symptoms showed little improvement. Clinical examination revealed the absence of the right maxillary third molar. Panoramic x-ray showed a tooth-like high-density shadow in the right maxillary sinus, surrounded by soft tissue density. Cone beam computed tomography (CBCT) further confirmed the presence of an ectopic third molar in the right maxillary sinus, accompanied by a cystic lesion. The patient underwent surgical removal of the ectopic tooth and complete excision of the cyst under general anesthesia via an intraoral approach. Postoperative histopathological examination confirmed the diagnosis of odontogenic keratocyst. The patient's symptoms completely resolved after surgery, and a 6-month postoperative CBCT review showed good pneumatization of the right maxillary sinus with no signs of recurrence.

**Conclusion:**

For persistent unilateral sinus symptoms that are unresponsive to conventional medical treatment, the presence of an ectopic maxillary third molar with an odontogenic cyst should be considered. Good clinical outcomes can be achieved by surgically removing the ectopic tooth and the associated cyst. This case also suggests that when tooth eruption is abnormal, early imaging evaluation is of significant value.

## 1 Introduction

An ectopic tooth is defined as a tooth that forms or erupts from a position other than the anatomic location to which it originally belonged through deviation from its path of physiologic eruption (1). Ectopic teeth present in atypical locations such as the nasal cavity, orbital floor, and maxillary sinus are noted. While the maxillary canines are the most commonly reported ectopic teeth, ectopic teeth found in the maxillary sinus are rare and are typically the third molars (2, 3).

Ectopic maxillary sinus teeth may be accidentally found during radiographic evaluation, but some may be incidentally associated with odontogenic cysts and can be symptomatic (4). Clinical features are often non-specific, with nasal obstruction being unilateral, nasal purulence, facial pressure or pain, recurrent swelling of the face, and maxillary sinusitis symptoms (5, 6). Due to overlapping symptoms with inflammatory disease, diagnosis can be impeded.

The case presents an ectopic maxillary third molar in the maxillary sinus with an odontogenic keratocyst in a young adult female. The clinical presentation, imaging, surgical aspects, histopathological aspects, and follow-up result of the case describe the case from its onset to the final clinical outcome, highlighting the utility of CBCT and the relevance of the odontogenic origin of cases where unilateral sinus signs cannot be managed. Despite the subject being an adult, the case has applicability in pediatric dentistry regarding its pedodontic presentation demonstrating the nature of its eruption disturbances and the necessity of early evaluation using imaging in cases of failure to find an eruption site in the expected position.

## 2 Case report

A 24-year-old female was referred to the office for presentation of purulent nasal discharge and recurrent swelling in the right maxillary quadrant for 2 years. The patient had been treated previously for 2 years with antibiotics and other medications for suspected rhinosinusitis at another outside hospital, but to no avail. Past medical history was negative with no facial trauma or history of extraction of the right maxillary third molar. Clinical examination showed normal facial symmetry. Mouth opening and mentation were normal. Intraoral examination was normal, and assessment found all permanent teeth to have erupted except the right maxillary third molar. The buccal and palatal mucosa in the adjacent maxillary quadrant were normal with no evidence of swelling and fluctuation. Palpation along the right maxillary region was normal and revealed mild tenderness.

Panoramic radiography revealed a well-defined dentally situated high-density shadow with a tooth-like appearance in the right maxillary sinus surrounded by soft tissue density shadows. No third molar was depicted distal to the second molar, suggesting its ectopic eruption into the right maxillary sinus (Figure 1). CBCT revealed a fully erupted ectopic third molar in the right maxillary sinus. The tooth crown extended into the sinus cavity. The roots of the tooth were located next to the medial wall of the sinus (Figure 2). A cystic soft tissue density lesion surrounded the tooth and the anterior, posterior, and floor of the maxillary sinus (Figure 2). Clinically and on imaging examination, the provisional diagnosis of ectopic right maxillary third molar with odontogenic cyst in the maxillary sinus of the right side was reached, and surgical treatment under general anaesthesia was indicated.

**Figure 1:**
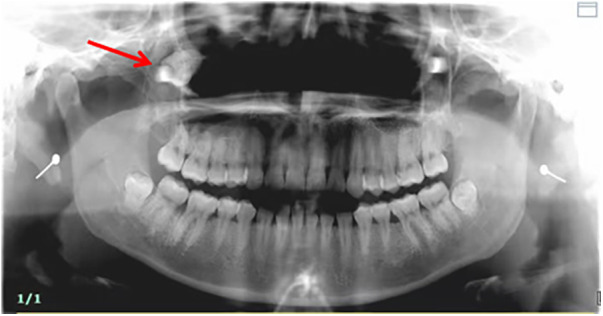
Preoperative panoramic radiograph showing a tooth-like radiopaque lesion (arrow) in the right maxillary sinus, suggestive of an ectopic tooth.

**Figure 2:**
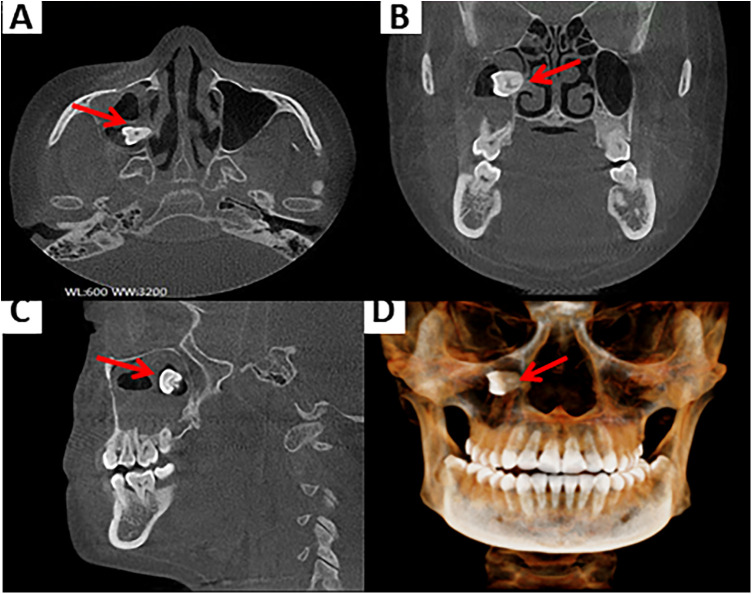
Preoperative CBCT images of the right maxillary sinus. **(A)** Axial, **(B)** coronal, and **(C)** sagittal views demonstrating the ectopic third molar (arrow) within the sinus cavity, surrounded by a cystic hypodense lesion. **(D)** Three-dimensional reconstruction highlighting the spatial relationship between the ectopic tooth, the cystic lesion, and the maxillary sinus anatomy.

After nasotracheal intubation, a vestibular incision was raised from the first premolar to the molar region and a mucoperiosteal flap was reflected with elevation of the anterior wall of the maxillary sinus. A rectangular bony window cavity was prepared in the front wall using a piezosurgery instrument. On entering the sinus bone cavity, a cystic lesion having an ectopic tooth as its content was identified. The lesion was dissected out and completely removed, and then the ectopic tooth was loosened and extracted (Figure 3). The sinus cavity was properly irrigated with normal saline. White keratinized debris and inflammatory secretions were identified intraoperatively and thoroughly removed. On suspicion of the lesion being an odontogenic keratocyst, freshly prepared Carnoy's solution was applied to the surgical cavity for chemical cauterization. Following hemostasis, drainage strips were placed and the flap was repositioned and sutured. Postoperative management included a course of antibiotic therapy, analgesia, edema control, and nasal care (avoid forceful nasal blowing; nasal saline was used as needed). Histopathological examination confirmed that the cyst wall was lined with keratinized stratified squamous epithelium with a basal cell layer consisting of columnar cells showing a palisading arrangement and supporting the odontogenic keratocyst diagnosis (Figure 4).

**Figure 3:**
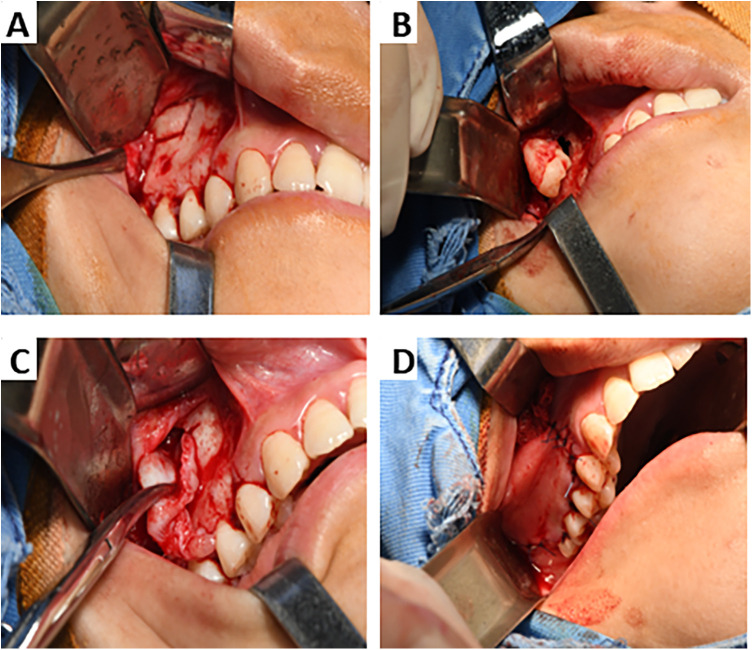
Intraoperative photographs. **(A)** Exposure of the anterior wall of the right maxillary sinus via a vestibular incision and creation of a bony window using piezoelectric surgery. **(B, C)** The extracted ectopic third molar (arrow) and the surrounding odontogenic keratocyst after complete enucleation of the lesion. **(D)** The extracted ectopic tooth and the surgical site after thorough irrigation and hemostasis.

**Figure 4:**
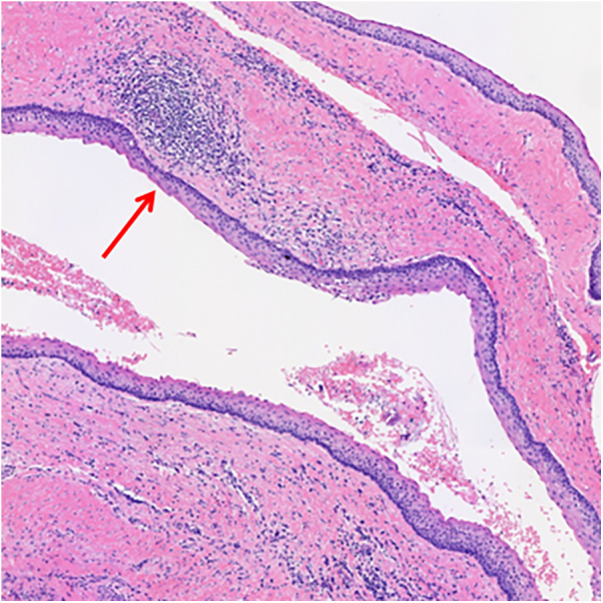
Histopathological image of the lesion (H&E, ×100) showing a cyst wall lined by keratinized stratified squamous epithelium with a palisaded basal layer (arrow), consistent with odontogenic keratocyst.

Purulent nasal discharge decreased gradually until totally absent. The trans-oral (premolar region) incision healed well (Figure 5). Following 3-month postoperative follow-up with CBCT, there was aeration of the right maxillary sinus with no remaining dentition or cyst recurrences (Figure 6). The patient reported total eradication of the symptoms and substantial quality of life improvement.

**Figure 5:**
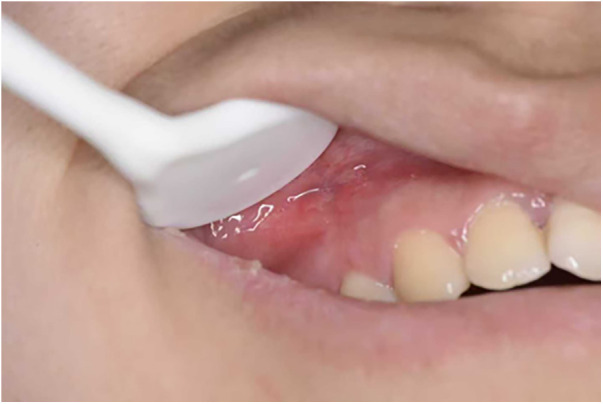
Postoperative intraoral photograph obtained 1 month after surgery, showing satisfactory healing of the surgical site.

**Figure 6:**
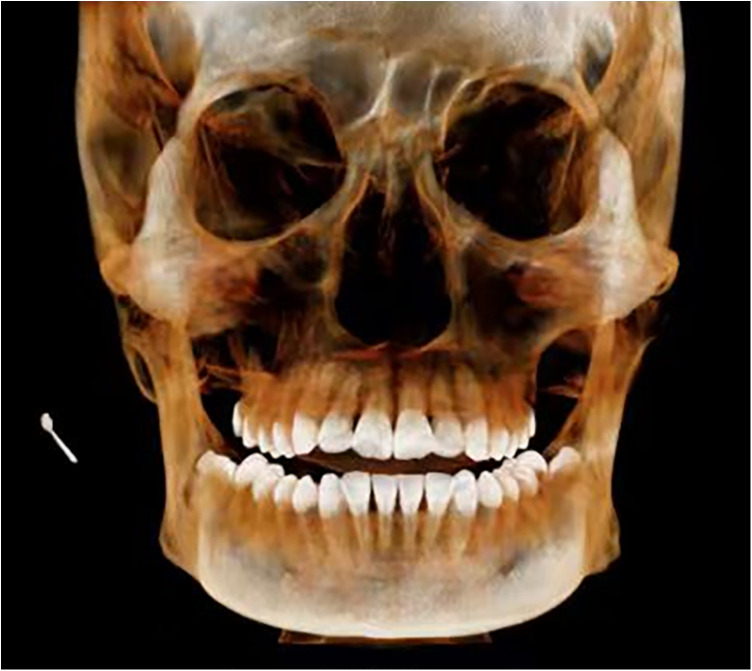
Postoperative CBCT at 6-month follow-up showing restored aeration of the right maxillary sinus and no evidence of recurrence.

## 3 Discussion

Ectopic teeth within the maxillary sinus are rare, but the third molars are the teeth most affected. The etiology is complex, but it is thought to be related to an abnormal development of the tooth germ, an altered odontogenic trajectory, displacement caused either by odontogenic cysts or tumors, bone resorption associated with an infection or trauma, or embryologic factors, or genetic factors (2, 7). The lesion is often asymptomatic and can only be incidentally detected with radiographic evaluation. Symptoms, when they do present, are non-specific and can resemble chronic rhinosinusitis and include unilateral nasal obstruction, nasal purulent discharge, facial pain or pressure, facial edema, headache, and recurrent sinusitis (7, 8). In contrast to several previously reported cases in which ectopic maxillary sinus molars remained asymptomatic and were discovered incidentally (2), the present patient suffered from refractory unilateral purulent rhinorrhea and recurrent facial swelling over two years, symptoms that had been repeatedly misattributed to infectious rhinosinusitis—a clinical scenario more akin to that reported by Srinivasa Prasad et al. (8), but differing from the bilateral presentation described by Berberi et al. (7). This underscores that even unilateral, subtly presenting dental anomalies can underlie chronic sinus complaints that fail medical therapy. Clinical history supports the notion that the underlying cause of his symptoms had not been addressed. There was no clinically detectable right maxillary third molar, and radiographic imaging demonstrated the presence of a tooth-like high-density material within the maxillary sinus, which was key in identifying the odontogenic origin of the lesion.

Panoramic radiography may aid as a first radiographic examination; however, the superimposition of overlying structures may hinder the accurate localization. CBCT has a particular advantage in the visualization of the ectopic tooth in three dimensions by showing the relation of its orientation to the sinus walls, the extent of the development of the roots, and the extent of the associated lesions. In this instance, CBCT was necessary both for the confirmation of the diagnosis and for the planning of the surgical approach (9). While Elmorsy et al. (2) highlighted CBCT in delineating an infected dentigerous cyst associated with an ectopic molar, the present case further illustrates CBCT's value in differentiating odontogenic keratocysts from the more frequently encountered dentigerous cyst in this anatomical setting. Preoperatively, the differential diagnosis primarily centered on distinguishing between these two entities. Unlike typical dentigerous cysts that usually exhibit a well-defined, unilocular radiolucency tightly adherent to the cementoenamel junction, the present cystic lesion displayed a more extensive involvement of the sinus floor and demonstrated subtle internal density variations on axial slices. Although radiographic features alone are insufficient for a definitive diagnosis, the lack of a clear “dependent” relationship between the cyst wall and the tooth crown, coupled with the patient's refractory symptoms despite antibiotic therapy, lowered the suspicion for a simple inflammatory dentigerous cyst and raised the possibility of an odontogenic keratocyst or other neoplastic processes. In this case, it was evident that there was an ectopic tooth projecting into the sinus cavity with roots closely related to the sinus walls. The radiographic triad allows differentiation of inflammatory sinus disease, retention sinus cysts, fungal sinusitis, benign odontogenic lesions, and tumours of the maxillary sinus. Nevertheless, a tooth-like high-density shadow in the maxilla is a strong argument for diagnosing an ectopic tooth and the definitive diagnosis of any associated lesion requires histopathology (10). In this case, the definitive diagnosis was an odontogenic keratocyst. The indication for surgical removal of the ectopic tooth in the maxillary sinus is when the lesion is symptomatic, associated with a cyst, recurrent infection, or other complications; the type of surgical exposure should be individualized based on the position of the tooth, extent of the lesion, sinus anatomy, experience of the surgeon, and the need for curettage or enucleation of the cyst to obtain a cure. Compared with Ramishvili et al. (11), who employed a combined intraoral–endoscopic approach for a large dentigerous cyst in the maxillary sinus, the current case was managed successfully via a purely intraoral route, suggesting that minimally invasive transoral excision remains adequate for selected ectopic molars when the cyst is moderate in size and the sinus anatomy permits complete enucleation. However, it must be acknowledged that a purely intraoral approach may limit direct visualization of the superior and posterior sinus recesses compared to endoscopic assistance. In this specific case, the ectopic tooth and the bulk of the cyst were located anteriorly and inferiorly within the sinus, making them readily accessible via the Caldwell-Luc approach without the need for additional nasal instrumentation. The transoral approach offers direct surgical access, a wide operative field, and is advantageous if the ectopic tooth is deeper in the maxilla or if curettage or enucleation of the cyst is required. On the other hand, the transnasal endoscopic approach is less invasive and may minimize postoperative sequelae; however, it lacks widespread experience, requires preoperative evaluation, and is not appropriate in all situations (11).

Histological hallmark features of odontogenic keratocysts are the thin layer of keratinised stratified squamous epithelium often with a corrugated surface and palisaded basal cell layer. The keratinised layer is sometimes shed, producing keratin debris inside the cyst cavity. The clinical significance of this lesion lies in its potential for locally aggressive growth and known potential for recurrence. The reported recurrences for odontogenic keratocysts vary, although recurrence is said to be due to a thin epithelial wall, daughter cysts and incomplete extirpation (12). Complete enucleation should be advised with careful follow-up. Although no recurrence was observed within 6 months' follow-up of this case, follow-up should still be continued.

However, it must be acknowledged that a purely intraoral approach may limit direct visualization of the superior and posterior sinus recesses compared to endoscopic assistance. In this specific case, the ectopic tooth and the bulk of the cyst were located anteriorly and inferiorly within the sinus, making them readily accessible via the Caldwell-Luc approach without the need for additional nasal instrumentation. Furthermore, regarding the use of Carnoy's solution, while effective in reducing recurrence by devitalizing residual epithelial cells, caution must be exercised regarding potential damage to the normal maxillary sinus mucosa and impairment of sinus function. Considering the long-term perspective, performing a middle meatal antrostomy on the affected side could be a valuable adjunct in similar cases to facilitate postoperative drainage and endoscopic surveillance.This study has certain limitations inherent to its design as a single case report. The follow-up period of six months, although demonstrating initial success, is relatively short for monitoring odontogenic keratocysts, which are known for late recurrences; thus, long-term surveillance is imperative. As a retrospective analysis, the study lacked standardized pre- and post-operative quality-of-life metrics to objectively quantify symptomatic improvement.

Finally, there is particular pedodontic interest in this case, as the ectopic eruption of permanent teeth is, in fact, a type of developmental anomaly. Earlier periods during adolescence are available for the earlier diagnosis and treatment of these types of anomalies before the emergence of long-term sinus symptoms. For this reason, this report points out that appropriate and timely imaging assessment must be carried out and interdisciplinary collaboration between paediatric dental specialists, oral and maxillofacial surgical specialists, and otolaryngeal specialists is of high priority when unilateral sinusal symptoms coexist with an abnormal eruption pattern.

## 4 Conclusions

Ectopic maxillary third molars associated with odontogenic cysts are a rare but important clinical cause of persistent unilateral sinus symptoms. In patients with chronic or recurrent unilateral nasal discharge, facial swelling, or maxillary sinus symptoms that do not respond to medical treatment, an odontogenic cause should be considered. Good therapeutic outcomes can be achieved through appropriate imaging-based evaluation, selection of an appropriate surgical approach, and complete removal of the ectopic tooth and related lesions. This case also emphasizes the importance of early imaging assessment of missing or abnormally erupted teeth to prevent delayed diagnosis and long-term suffering.

### 4.1 Patient perspective

The patient reported complete resolution of her long-standing symptoms and expressed satisfaction with the surgical outcome.

## Data Availability

The original contributions presented in the study are included in the article/Supplementary Material, further inquiries can be directed to the corresponding author.
